# One Health approaches to improve refugee health

**DOI:** 10.1016/S2214-109X(21)00416-2

**Published:** 2021-12

**Authors:** Indorica Sutradhar, Muhammad H Zaman

**Affiliations:** Department of Biomedical Engineering, Boston University, Boston, MA 02215, USA; Department of Biomedical Engineering, Boston University, Boston, MA 02215, USA; Howard Hughes Medical Institute, Boston University, Boston, MA 02215, USA

By estimates from 2020, there are approximately 26·4 million refugees worldwide today and the refugee population has been increasing over the past decade.^1^ The unfolding situation in Afghanistan following the US withdrawal is likely to further increase the number of individuals who are forcibly displaced. The process of migration can expose refugees to risk factors for disease and hinder access to basic health care. Additionally, refugees often face continued disadvantage compared with host populations due to xenophobia and shortage of cohesive support in their host country.^2^ Infectious disease is of particular concern in refugee camps. Increased occurrence, severity, and case fatality for infectious diseases can occur in refugee camps due to factors including exposure during migration, poor sanitation conditions, shortage of vaccinations, and overcrowding.^2,3^ In fact, indirect causes, particularly infectious disease, account for the majority of deaths in conflict and displacement.^3^ Furthermore, refugees can also face treatment interruption, which can increase the risk of developing a drug resistant infection.^2^ However, interventions aimed at improving refugee health, such as vaccinations, are often only focused on human health factors. To fully address the challenges of refugee health, a broader scope when planning interventions could be necessary.

Although the effects of refugee camps on their environment have been the subject of several studies, the effects of environment on refugee communities, such as environmental contamination in sewage and wastewater, have received less attention. This shortage of research is troubling as sewage and wastewater can host dangerous pathogens, leading to disease in surrounding communities. Furthermore, it has been shown that wastewater is a reservoir for antimicrobial resistance due to the collection of antibiotic waste from humans and animals.^4^ Refugee camps often have open sewer systems and little access to wastewater treatment, putting people at particular risk of deadly drug-resistant outbreaks. Other environmental threats such as overcrowding and poor housing conditions have also been associated with illness in refugee camps.^5^ For instance, poor ventilation, moulds, and crowding were shown to be directly related to the incidence of upper respiratory tract disease in the al-Amar’ri refugee camp in the West Bank of Palestine.^6^ Another source of infection is contaminated drinking water as observed with rotavirus in a camp in Jordan.^7^ Conflict and weapons of war also affect the environment and enable the development and proliferation of drug resistant pathogens.^8^

Interactions between humans and animals are often ignored in the discussion of health of refugees, but animal populations are often closely tied with refugee communities. Many refugee communities rely on livestock to aid in self-sufficiency in food acquisition. For example, the Sahrawis in Western Sahara depend on a large population of goats, sheep, and camels.^9^ However, livestock zoonoses are widely occurring in low-income and middle-income countries (LMICs), which often host these refugee populations.^10^ Animals that migrate with refugee communities can contract new infections that they do not have immunity towards. Pests can also spread infection in refugee camps, in which they are often drawn to inappropriately disposed of waste.^5^ The risks presented by zoonoses are particularly of concern as they occur frequently but are understudied, and mismanagement of related cases can lead to a cycle of poor health in these communities.

These often neglected environmental and veterinary risk factors show the importance of taking a One Health approach to the challenges in refugee health. One Health is defined as approaching issues of global health by looking at the areas of human, animal, and environmental health, and their intersections (appendix). This approach can allow for the development of more effective interventions and preventive measures. These One Health approaches have been used successfully in LMICs; for example, in Latin America mass dog vaccination for rabies has been shown to be effective in preventing deaths in humans caused by rabies.^10^ Preventive measures such as these described can extend beyond rabies and apply to other zoonoses in which vaccines are available.

Developments in Afghanistan will increase the global refugee population and many will be forced to live in camps or poorly structured urban settlements. The threats to refugee health from animal and environmental sources cannot be ignored. Addressing these issues can allow society to address challenges in refugee health and prevent future crises. One Health offers potential systematic-level approaches for the health of forcibly displaced communities. Initial approaches could include previously successful interventions including animal vaccinations and wastewater management. Other interventions can be developed through further research, rooted in improved surveillance, data analytics, bioinformatics, and metagenomics, which can provide insights into disease dynamics and ethical and evidence-based interventions. Taken together, we believe that One Health approaches could provide an opportunity to address challenges of refugee health by considering not only the human variables, but also the animal and environment considerations in these communities.

## Supplementary Material

Supplementary Appendix
