# Supplementary material for: One Health approaches to improve refugee health
Source: Lancet Glob Health. Author manuscript; Available in PMC 2023 Jul 10. (PMC10332798; doi:10.1016/S2214-109X(21)00416-2)
Supplement: Supplementary Appendix [file NIHMS1907255-supplement-Supplementary_Appendix.pdf]

THE LANCET
Global Health

Supplementary appendix

This appendix formed part of the original submission. We post it as supplied by the authors.

Supplement to: Sutradhar I, Zaman MH. One Health approaches to improve refugee health. *Lancet Glob Health* 2021; **9:** e1646–47.

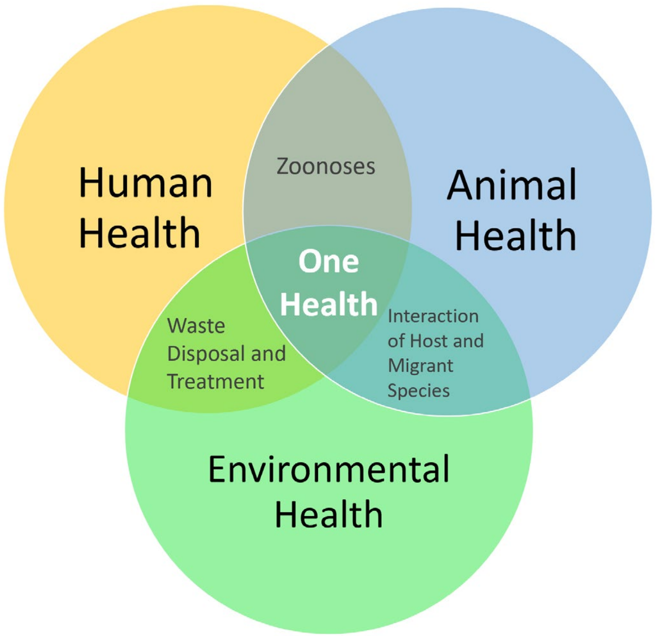

Figure: **Schematic representation of the One Health approach**
